# Oral Microbiome and Dental Caries Development

**DOI:** 10.3390/dj10100184

**Published:** 2022-09-30

**Authors:** Josie Shizhen Zhang, Chun-Hung Chu, Ollie Yiru Yu

**Affiliations:** Faculty of Dentistry, The University of Hong Kong, Hong Kong SAR, China

**Keywords:** dental caries, oral microbiome, oral microbiology, cariogenic bacteria, precision dentistry

## Abstract

Dental caries remains the most prevalent oral disease worldwide. The development of dental caries is highly associated with the microbiota in the oral cavity. Microbiological research of dental caries has been conducted for over a century, with conventional culture-based methods and targeted molecular methods being used in order to identify the microorganisms related to dental caries. These methods’ major limitation is that they can identify only part of the culturable microorganisms in the oral cavity. Introducing sequencing-based technology and bioinformatics analysis has boosted oral microbiome research and greatly expanded the understanding of complex oral microbiology. With the continuing revolution of molecular technologies and the accumulated sequence data of the oral microbiome, researchers have realized that microbial composition alone may be insufficient to uncover the relationship between caries and the microbiome. Most updated evidence has coupled metagenomics with transcriptomics and metabolomics techniques in order to comprehensively understand the microbial contribution to dental caries. Therefore, the objective of this article is to give an overview of the research of the oral microbiome and the development of dental caries. This article reviews the classical concepts of the microbiological aspect of dental caries and updates the knowledge of caries microbiology with the results of current studies on the oral microbiome. This paper also provides an update on the caries etiological theory, the microorganisms related to caries development, and the shifts in the microbiome in dental caries development.

## 1. Introduction

Dental caries is the most common oral disease worldwide. Untreated caries affects 2.5 billion adults and 573 million children all over the world [1], placing a heavy health burden on health care systems and society. Over the past 25 years, the prevalence of dental caries has remained at a similarly high level despite oral health care providers’ efforts [2]. The high prevalence of dental caries indicates the effect of the research on dental caries. Dental caries is a multifactorial disease that involves microbial, behavioral, genetic, and environmental factors [3]. Although these factors are important in caries development, the role of microbial factors cannot be ignored. Because the development of dental caries is closely correlated to oral microorganisms, a comprehensive understanding of caries microbiology is essential.

The microbiology of dental caries has been investigated for over a century, with a revolutionized advance in study approaches. Previous studies have used traditional culture-based methods in order to identify the bacteria related to dental caries [4]. The culture-based method has allowed for a basic understanding of the dental plaque microbiota composition in dental caries to be established. Microorganisms have been isolated from carious lesions or dental plaque samples collected from a cross-sectional or longitudinal study using culture-based techniques [5,6]. However, bacteria can only be successfully cultured when provided with their own special growth requirements. At present, because artificial media cannot exactly mimic the natural environment within which oral bacteria reside, most fastidious bacteria remain uncultivable in vitro [7].

Targeted molecular methods for identifying and quantifying caries-associated bacteria were introduced in the early 1990s [8,9]. Targeted molecular methods use DNA probes obtained from cultured bacterial species to enumerate the bacteria [10]. This allows for identifying and quantifying multiple species in dental plaque samples from more individuals compared to culture-based methods. This also facilitates the analysis of species that are difficult to culture [4]. However, targeted molecular methods can only detect the preselected bacteria that culture-based methods have already confirmed. Unknown bacteria sets in the dental plaque cannot be detected.

The introduction of sequencing-based technology and bioinformatics analysis has greatly expanded the understanding of complex oral microbiology. Unlike the targeted molecular methods, next-generation sequencing (NGS) techniques identify sequences by comparing them to the curated microbiome database, enabling the detection of novel species that have historically been uncultivable [11]. Since the development of the Sanger sequencing technique in 1977, great efforts have been made to conquer the high cost and low efficiency of “reading” genes. NGS, also known as massively parallel sequencing technology, stands out for allowing ultra-high throughput with a deeper coverage of the microbial community at a lower cost. During the last decade, targeted 16S rRNA amplicon sequencing, which relies on the amplification of one to three selected hypervariable regions of 16S rDNA, has been widely adopted to study the oral microbiome’s compositional changes in dental caries. However, sequencing fragments of the 16S rRNA gene can miss variants that discriminate different species or strains, which compromises this technique’s taxonomic resolution [12].

The improvement in sequence length in metagenomics overcomes the limitation of 16S rRNA gene sequencing. It is promising in identifying more members of the microbial community [13]. Studies that are more recent coupled metagenomics with transcriptomics and metabolomics to investigate how the active microbial community collaborates in the initiation and progression of dental caries [14,15,16]. They investigated what the microorganisms in the studied niches were, as well as how the microorganisms actively worked together to cause disease. The understanding of caries microbiology has been greatly enriched with the advancement of sequencing-based technology and bioinformatics analysis in recent years. Therefore, this article aims to review the classical concepts on the microbiological aspect of dental caries and to update the knowledge of caries microbiology with the results of studies on the oral microbiome conducted over the past few years.

## 2. Materials and Methods

**Figure 1:**
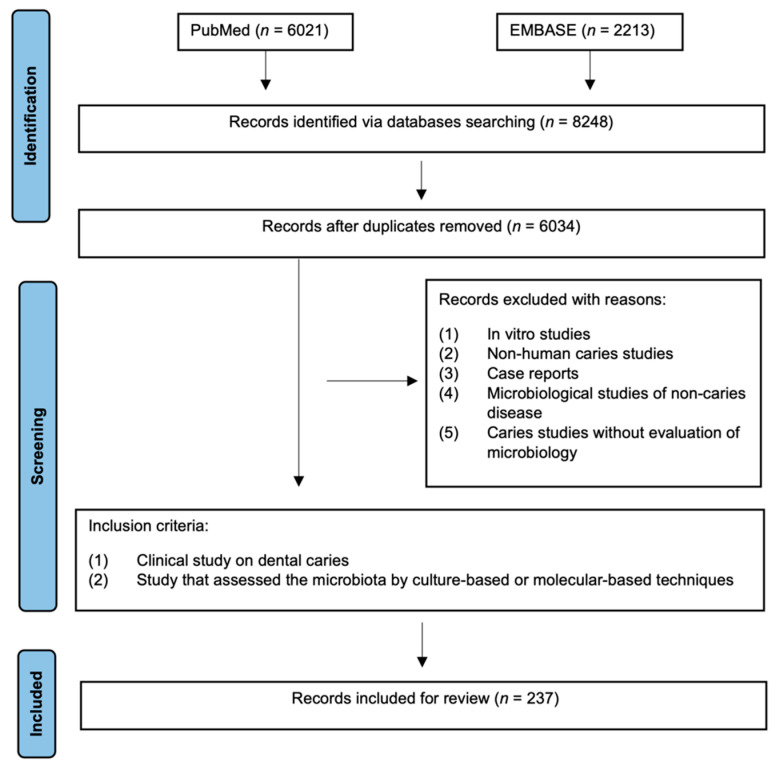
Flow diagram of literature search and study selection.

We searched the two most relevant electronic databases, PUBMED and EMBASE, for published evidence with the combination of the following key words: (caries OR “dental decay” OR “tooth decay” OR “carious lesion” OR “white spot”) AND (microbiome OR microbiota OR microbial OR biofilm OR microorganism OR mycobiome OR virome). Studies were limited to English publications published on or before 1 August 2022. Duplicate studies were discarded. Studies were included for review if they met the following criteria: (1) a study on human dental caries or (2) a study that assessed the microbiota of caries using culture-based or molecular-based techniques. The study selection process is presented in the flow diagram below (Figure 1).

## 3. Results

### 3.1. Oral Microbial Communities

The oral cavity is a complex ecology with various niches. Not only bacteria but also archaea, fungi, and viruses reside in these niches [17,18]. Among them, bacteria make up the main proportion of this diverse community. Bacteria are also the most extensively studied subtype of the oral microbiome. According to the expanded Human Oral Microbiome Database (eHOMD) (https://homd.org/ (accessed on 1 August 2022)), 774 oral bacterial species have been detected and studied. Around 58% of the species have been cultivated and officially named, 16% have been cultivated but remain unnamed, and 26% remain uncultivated. The majority of these species belong to six broad phyla: Firmicutes, Actinobacteria, Proteobacteria, Fusobacteria, Bacteroidetes, and Spirochaetes [19].

For several decades, cultivation studies have reported fungi to be oral inhabitants [20]. Their biodiversity and potential role in the oral ecosystem could not be extensively studied until the last decade because of the uncultivable features of most fungi and the relatively low proportion of the biomass [21]. A collection of studies has found more than 100 genus-level taxa of fungi as significant constituents of the oral mycobiome, of which only Candida and Malassezia have been well demarcated [22,23,24]. Moreover, only Candida has been extensively investigated and claimed to be involved in various oral diseases, including caries [25,26]. Although studies have reported hundreds of fungal taxa, only a few of them have been indicated to be true oral colonizers [22]. The further isolation and characterization of the other fungal taxa with low abundances are desirable for understanding the diversity and functionality of the fungi in the oral microbiome.

The Archaeal microorganism was first isolated from an oral subgingival sample in 1987 [27]. Later studies found the presence of Archaea at various oral sites, most frequently at periodontal sites [28,29,30,31,32,33], followed by endodontic sites [34,35], but rarely from dental caries [36], saliva [37], and the tongue [38]. Currently, Archaea detected in the human oral cavity is confined to a few phylotypes, including the most abundant Methanogenic archaea and Thermoplasmata [36,39,40]. The low abundance and the fastidious cultivation process can hamper the identification of Archaea, which can lead to an underestimation of the diversity of oral archaea. Furthermore, the information related to the role of oral archaea in dental caries is sparse.

Viruses have been identified from oral cavities, including a few eukaryotic viruses and various bacteriophages [41]. Interpreted from the limited evidence, the oral virome is highly individual-specific but temporally stable [42,43]. Bacteriophages, which are viruses targeting bacteria, are primarily lysogenic. In a previous study, the bacteriophages of lytic styles, which were predominant in the dental plaque of periodontitis, could eradicate their susceptible bacteria hosts or convey new functions to their bacteria host [44]. Thus, viruses may have a considerable capacity in shaping the oral microbial community’s structure and pathogenesis [45]. Despite the significance of virus–bacteria interactions to the whole oral microbiome, the role of oral viruses is understudied [45]. The interspecies and inter-kingdom interactions of the oral microorganisms are the key to maintaining oral health. The pathogenic shifts in the oral microbial communities contribute to the pathogenesis of polymicrobial diseases, including caries [33,44,46,47]. Therefore, the development of caries is highly associated with oral microbiological changes [2,14].

### 3.2. The Microbiological Hypothesis of Dental Caries Etiology

The continuous development of microbiological research methods has led to a shift in the microbiological theory of dental caries etiology. In the late nineteenth century, bacteria isolation and identification techniques were far from developed. The etiology of caries was postulated to be determined by the quantity of dental plaque, referred to as the “Traditional Non-specific Plaque Hypothesis”, which Miller proposed in 1890 [48]. With advances in microscopes and microorganism cultivation techniques, specific bacterial species, mainly Streptococcus mutans and Lactobacillus, were frequently found to be associated with initiating caries, characterizing them as cariogenic species [49,50,51,52]. Antibiotic treatment that targeted these species reduced caries formation [53]. Based on this evidence, Loesche proposed the “Specific Plaque Hypothesis” in 1976 [54]. The “Specific Plaque Hypothesis” states that specific cariogenic bacteria in dental plaque, such as Streptococcus mutans and Lactobacillus, are responsible for dental caries. However, the “Specific Plaque Hypothesis” cannot explain the fact that Streptococcus mutans is absent from some carious sites. In addition, Streptococcus mutans is constantly detected on sound tooth surfaces, indicating that the presence of this species is neither sufficient nor necessary for initiating caries [55].

With other bacteria species isolated from caries, Marsh proposed the “Ecological Plaque Hypothesis” in 1994 [56,57]. In this hypothesis, it is stated that caries develops along with the disruption of microbial homeostasis under ecological stress, in which some pathogenic species outnumber health-related microorganisms. The ecological plaque hypothesis stresses the critical role of the interaction between the environment and bacteria. The development of molecular identification techniques has led to a continuous revision of the etiology of dental caries. Hundreds of not-yet cultivable oral phylotypes have been identified using gene-sequencing methods, suggesting that an increasingly diverse microflora might play a critical role in the ecological changes resulting in caries onset [58]. Furthermore, a metatranscriptomic analysis revealed a discrete gene expression profile of oral microflora from which genomics are disclosed, directing the focus to the microbes that are actively involved in caries development [59]. In 2008, Takahashi further extended the “Ecological Plaque Hypothesis” by incorporating the metabolism of plaque microorganisms. In this extended version, it is stated that the neutral microenvironment tilts to an acidic condition when acid production outweighs the base metabolites’ buffering capacity, followed by acid-induced selection and adaptation within the microflora. This process disrupts a healthy microbial community’s stability and leads to dental caries [60].

### 3.3. Microorganisms Associated with Caries Development

Table 1 presents the microbial species that are potentially related to caries based on the currently available literature. Streptococcus mutans has been considered a major pathogen of dental caries since 1971 [49,61]. Many other bacteria have been isolated from carious sites or have been found to feature distinctly throughout the process of caries development, and they have been roughly proposed to be related to caries. With the development of culture-independent identification technology, some fastidious fungi have also been identified to be caries-related (Table 1).

It is worth noting that, even though various species of Lactobacillus and Bifidobacteria were reported to be strongly correlated with caries progression, other species of these two genera were demonstrated to be effective probiotics in the context of caries prevention [90]. These probiotics may exert caries prevention effects by regulating the microflora dysbiosis induced by environment stress [91]. Considering that different species of the same genus may play opposite roles in the caries development process, a species-level resolution analysis is required [92].

With the evolutional sequencing-based technology and bioinformatics approaches, more unknown bacterial species were identified, and their roles in the acid-producing process were gradually disclosed [93]. A comprehensive understanding of caries microbiology is under development.

### 3.4. Shifts in the Oral Microbiome in Dental Caries

Evidence on the association between the oral microbial profile and caries has been surging in recent decades [94]. Generally, the oral bacterial community was less diverse in caries-affected than in caries-free subjects [95,96,97]. Specifically, the relative abundance of caries-related species rather than the taxonomic diversity changed along with caries development [83,94,98,99,100,101]. The changes in the fungal microbiome, or the mycobiome, were similar to those of the bacteriome, with the relative abundance of several taxa that mainly belong to candida increasing significantly in the dental plaque on caries surfaces [92]. The microbiome research of dental caries has not detected any specific microbial species uniquely associated with caries [102].

Different microbial interaction profiles between caries-affected and caries-free communities were found in an operational taxonomic unit (OTU) network analysis, although the results are discordant among studies. In a study on early childhood caries (ECC), the interconnection between OTUs was reported to be intensified in the caries-affected community compared to the caries-free community, suggesting the contribution of the intensive species interactions to the development of ECC [94]. In another study, the intercorrelation among predominant genera was increasingly complex and robust in caries-free sites in an adult population [101]. The research subjects’ different age groups may explain the inconsistency between the studies. Further explorations are required to obtain a valid conclusion on the changes in microbial interactions in patients with caries.

**Figure 2:**
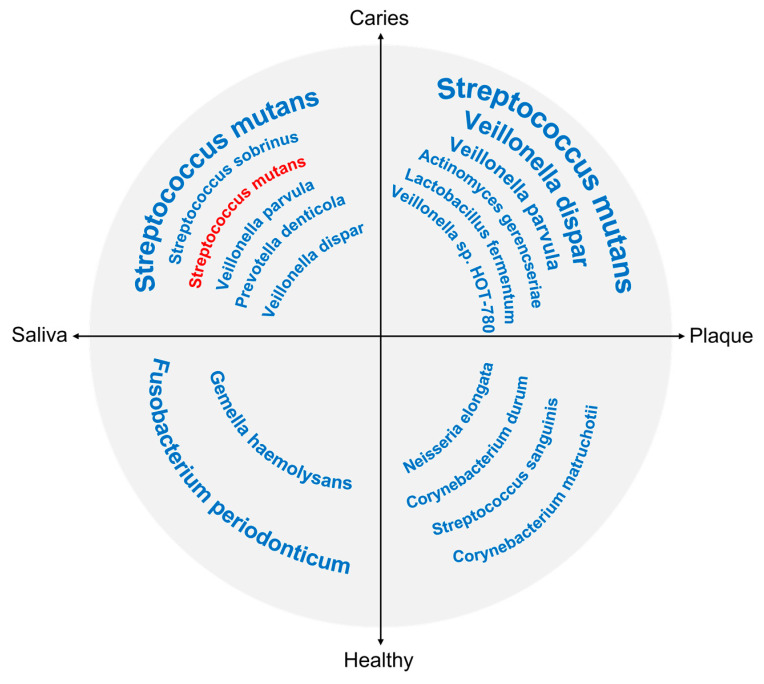
Common bacteria affecting development of dental caries. Bacterial species with significantly higher abundances in caries-affected status or in healthy status from supragingival plaque or saliva samples. Each quadrant presents a different sample type; for example, the top right quadrant denotes plaque samples from caries-affected subjects. Font size is positively proportional to the frequency of detection. Font color is to distinguish age groups: blue for children and red for adults.

Because microbiome studies on dental caries are carried out separately in children and adults, there is no direct evidence on comparing the difference in the caries-related microbial shift between different age groups. However, evidence has shown that the microbial composition changes with dentition development [103]. Thus, the microflora that contributes to the caries process may differ from childhood to adulthood. We extracted the bacterial species with a significantly higher abundance in caries-affected and caries-free subjects from previous articles, and they are presented in Figure 2. Considering repeatability, only species that were reported in at least two studies were included as being caries-related or health-related. As shown in Figure 1, only Streptococcus mutans from saliva was shown to be significantly associated with caries in adults, while a variety of taxa from both saliva and plaque were reported to be caries-related or health-related in children [102].

Apart from the overall changes in the oral microbiome in patients with dental caries, the shifts in the caries-associated microorganisms at the different statuses of caries progression were also investigated.

#### 3.4.1. Microbiome Shifts in Caries of Different Stages

The organic and mineral components, as well as the micro-environment, are diverse throughout the different parts of the tooth structure, indicating that the destruction of the different tooth parts may involve distinct microbial-related factors. An early metagenome study on caries reported that both the compositions and the functional profiles of the microbial communities differed greatly between enamel caries and dentin caries [97]. Genes that encode the functions of sugar fermentation, cell surface adhesion, and acid stress responses were overrepresented in enamel caries while presenting the opposite trend in dentine caries [97,104]. Interestingly, genes overexpressed in the deep dentin microflora correlated to the host’s immune response [97], indicating the potential significance of the interaction between microbes and their host. Additionally, Lactobacillus species were only detected in dentin caries, and a higher abundance of Prevotella was found deep in the dentin [97]. According to Richards et al., four species (*S. mutans*, *Scardovia wiggsiae*, *Parascardovia denticolens*, and *Lactobacillus salivarius*) exclusively exist in dentine caries [90]. On the contrary, some species with a higher abundance in enamel caries decreased or disappeared in dentine caries [97]. A metatranscriptomic study also showed that the active microbial community differed significantly between carious enamel and dentine, with lactobacilli expressing a much higher level in dentine caries [104]. In this study, a large number of species expressed at an extremely low abundance existed exclusively in either enamel caries or dentine caries, which indicates that minority species might play an essential role in caries occurrence [104].

#### 3.4.2. Microbiome Shifts in Caries with Different Activities

Recently, the microbial contribution to dental caries activity was investigated. It was concluded from the evidence that existed that the microbial communities residing on active caries saw a reduction in richness compared to those residing on arrested caries [105], but they shared a similar beta diversity [105,106]. Additionally, the relative abundance of some caries-associated bacteria was increased in resistant active caries after silver diamine fluoride (SDF) treatment but was decreased in arrested caries [105]. An in vivo study found that Streptococcus and Veillonella were more evenly distributed with other taxa in arrested caries, while they were predominant in induced active caries [107]. Further evidence is needed to investigate the microbiome’s role in the shift in caries activity.

#### 3.4.3. Microbiome Shifts in Caries in Different Locations

The biofilm composition of the dental root surface may differ from that of the coronal surface because of the influence of gingival crevicular fluid [108]. Patients with coronal caries may be free from root caries and vice versa. Thus, there arises the question of whether the bacterial community involved in root caries might differ from that involved in coronal caries. An early culture-based study showed that the major bacteria recovered from the plaque of root caries were distinct from those of enamel caries [109]. However, no molecular study has investigated the dis/similarity of the microbiome involved in coronal caries and root caries. Most of the up-to-date oral microbiome studies targeted the relationship between the microbial community and coronal caries. A recent study focusing on root caries found that Prevotella dominated the microflora of caries lesions that extended to the subgingival margin, while Streptococcus dominated the microbial community from root caries lesions that were confined within supragingival sites [110]. However, the periodontal microflora probably confounded the results of this study. The lack of evidence on this subject indicates the urgency of exploring this phenomenon.

## 4. Discussion and Future Perspectives

The recent advancements in oral microbiology studies have expanded our perspectives on caries etiology. Nevertheless, there are limitations in the caries microbiology studies discussed in the present literature review. Due to the high cost of the new-born omics techniques and the infancy of methodological and analytical protocols, most of the recent studies used 16S rRNA gene sequencing to investigate the microbiological contribution to caries development. However, 16S sequencing only provides information regarding bacterial composition at genus- or species-level resolution, failing to characterize the strain-level diversity of the microbial community and its functional capabilities [111]. Another non-negligible limitation is that current studies rarely study the diversity and the potential roles of nonbacterial microorganisms, regardless of the enormous diversity of the fungi identified in the oral cavity. As any other infectious disease, the host factor plays a vital role in caries development. Saliva, being the first line of defense in the oral cavity, helps shape the microbial profile of pioneer colonizers, which later “trains” the host immune system to defend against pathological invaders [112]. The dynamic balance between the host immune system and the microbial commensals maintains the oral health status [113]. Despite the convincing relationship between immunology and microbiology in the context of caries, there is a lack of studies examining how the microbiota interacts with the host immune system in the course of microbial dysbiosis. Lastly, the lack of consistency in the study protocol hampers the comparisons between studies, compromising the reproducibility of the results.

Future work should be directed toward resolving the limitations in the current studies. As demonstrated, the taxa detected with a high abundance in dental caries do not necessarily actively function and vice versa. Therefore, associating the microorganisms with their functional contributions to dental caries is highly desirable in future studies. Complementary methodological approaches are expected to be employed to uncover the diversity and contribution of the relatively unexplored domains of the microorganisms and their interactions with their bacteria counterparts in the oral microbiome community. Furthermore, the interaction between the microbial community and their host’s immune system should also be explored. Regarding the significant heterogeneity among the results of different studies, consistency in the study protocol, including sampling site selection, sampling methods, sample storage condition, sequencing technique, reference database, and bioinformatics pipelines, is required to achieve comparable results across the extensive number of studies.

## 5. Conclusions

Research using sequencing-based technology and bioinformatics analysis has revolutionized the classical microbiological concept of dental caries, which is based on culture-based methods. Contemporary evidence validates and extends the “Ecological Plaque Hypothesis”. The inter-species and inter-kingdom interactions of diverse microorganisms contribute to the development of dental caries. Both the predominance and the relative abundance of microbiota change along with the caries development stages. Researchers are further exploring the functionalities of caries-contributing species and the interactions between microbiota and their hosts.

## Figures and Tables

**Table 1:** Microbial species related to caries.

Microbial Species	Dentition	Location	Infected Tissue	Role in Caries	References
Bacteria					
*S. mutans*	Primary	Coronal	Enamel and dentine	Biofilm formation Caries initiation and progression	[62,63,64,65,66]
*S. sobrinus*	Primary	Coronal	N/A	N/A	[64,67,68,69,70,71]
*L. salivarius*	Primary and permanent	Coronal and root	Dentine	Caries progression	
*L. gasseri*	Primary and permanent	Coronal and root	Dentine	Caries progression	
*L. fermentum*	Primary and permanent	Coronal and root	Dentine	Caries progression	
*L. casei*	Primary and permanent	Coronal and root	Dentine	Caries progression	
*A. israelii*	Permanent	Root	N/A	Caries initiation and progression	[5,72,73]
*A. gerencseriae*	Primary and permanent	Coronal and root	N/A	Caries initiation and progression	
*A. naeslundii*	Permanent	Root	N/A	Caries initiation and progression	
*S. wiggsiae*	Primary	Coronal	Dentine	Caries progression	[67,69,74,75,76]
*P. denticolens*	Primary and permanent	Coronal and root	Dentine	Caries progression	[69,75]
*B. dentium*	Permanent	Coronal and root	Dentine	Caries progression	[74,75,76,77,78]
*B. longum*	Permanent	Coronal and root	Dentine	Caries progression	
*B. breve*	Permanent	Root	Dentine	Caries progression	
*L. shahii*	Primary	Coronal	N/A	Caries progression	[71,77,78,79,80,81]
*L. HOT 498*	Primary	Coronal	N/A	Caries progression	
*P. melaninogenica*	Primary	Coronal	Enamel and dentine	Caries progression	[77,78,79]
*V. dispar*	Primary	Coronal	Enamel and dentine	Biofilm formation Caries initiation and progression	[71,79,82,83]
*V. parvula*	Primary and permanent	Coronal and root	Enamel and dentine	Caries initiation and progression	
*V. denticariosi*	Primary	Coronal	Enamel and dentine	Caries initiation and progression	
Fungi					
*C. albicans*	Primary	Coronal	Enamel	Symbiotic with *S. mutans* Caries initiation	[79,84,85,86,87,88,89]
*C. dubliniensis*	Primary	Coronal	N/A	Caries progression	
*N. oryzae*	Primary	Coronal	N/A	N/A	[89]

## Data Availability

Not applicable.
